# Comparative mitogenomes provide new insights into phylogeny and taxonomy of the subfamily *Xenocyprinae* (Cypriniformes: Cyprinidae)

**DOI:** 10.3389/fgene.2022.966633

**Published:** 2022-10-25

**Authors:** Zhi Zhang, Jiming Li, Xiaodong Zhang, Bingming Lin, Jianming Chen

**Affiliations:** ^1^ Fujian Key Laboratory on Conservation and Sustainable Utilization of Marine Biodiversity, College of Geography and Oceanography, Minjiang University, Fuzhou, China; ^2^ Longyan Fisheries Technology Extension Center, Longyan, China; ^3^ Liancheng Jiming Fish Farming Company, Longyan, China

**Keywords:** *Xenocyprinae*, mitogenome, phylogeny, divergence time, taxonomy

## Abstract

*Xenocyprinae* is a cyprinid subfamily that not only has a discrete geographic distribution but also has a long history dating to the Early Miocene. However, it is controversial whether systematic classification and some species validity of *Xenocyprinae* exist, as well as its phylogenetic relationships and evolutionary history. In the present study, we first reviewed the description and taxonomic history of *Xenocyprinae*, and then the complete mitochondrial genome of *Distoechodon compressus*, an endemic and locally distributed species belonging to *Xenocyprinae*, was sequenced and annotated. Finally, all the mitogenomes of *Xenocyprinae* were compared to reconstruct the phylogenetic relationship and estimate the divergence time. The results showed that the mitogenomes are similar in organization and structure with 16618–16630 bp length from 12 mitogenomes of eight species. Phylogenetic analysis confirmed the monology of *Xenocyprinae* and illustrated three clades within the *Xenocyprinae* to consist of ambiguous generic classification. *Plagiognathops* is a valid genus located at the base of the phylogenetic tree. The genus *Xenocypris* was originally monophyletic, but *X. fangi* was excluded. Divergence time estimation revealed that the earliest divergence within *Xenocyprinae* occurred approximately 12.1 Mya when *Plagiognathops* separated from the primitive *Xenocypri*s. The main two clades (*Xenocypris* and (*Distoechodon* + *Pseudobrama* + *X. fangi*)) diverged 10.0 Mya. The major divergence of *Xenocyprinae* species possibly occurred in the Middle to Late Miocene and Late Pliocene, suggesting that speciation and diversifications could be attributed to the Asian monsoon climate. This study clarifies some controversial issues of systematics and provides essential information on the taxonomy and phylogeny of the subfamily *Xenocyprinae*.

## Introduction

*Xenocyprinae* is a cyprinid subfamily that not only has a discrete geographic distribution (restricted to East Asia, especially in China) but also has a long history (its extant genera and species became dominant and continued to develop in the beginning of the Early Pliocene) (Chang et al., 1996). The subfamily *Xenocyprinae* made it possible to distinguish populations and species in response to both profound and more recent historical environmental changes in East Asia (Xiao et al., 2012). Moreover, many of the species have been domesticated into excellent aquacultural varieties with high economic value (Lin et al., 2021), and they could also be used for ecological restoration of rivers to improve water quality and control the algae bloom in freshwater because most of them graze on benthic algae and debris (Zhang et al., 2015). Hence, the taxonomic status, historical evolution, and phylogeny of *Xenocyprinae* have become subjects of increasing concern.

The subfamily *Xenocyprinae* was established by Günther (1868) and subsequently discussed in terms of systematics and phylogeny. With further studies on the systematic status, different views on its classification have emerged. It appeared to be similar to *Chondrostoma* because of the sharp horny beak of the lower jaw and six main teeth of the lower pharyngeal teeth (Chu, 1935; Yang, 1964). However, Howes (1981) gave a contrary report because the superior pharyngeal process handle of the basal occipital was different, with many different signs and no overlap in distribution. Chen et al. (1984) incorporated *Hypophthalmichthyinae* into *Xenocyprinae* based on skeletal characteristics. The latest version of fishes of the world also supported that *Xenocyprinae* included *Xenocypris*, *Hypophthalmichthys,* and *Aristichthys*. In the current classification, *Fauna Sinica* is proposed to be a *Xenocyprinae*, including 10 species in four genera, as a sister group to *Hypophthalmichthyinae* (Liu, 1998). Zhao et al. (2009) reviewed them and proposed 12 species (previously 13, but *Distoechodon multispinnis* later seemed to be a subspecies of *D. tumirostris*) in four or five genera, which are the current systematics of *Xenocyprinae*. Molecular evidence of the subfamily *Xenocyprinae* is consistent with the results of previous studies (He et al., 2004; Tang et al., 2013), and the two main clusters and the genus *Xenocyprioides* are at the base of the phylogenetic trees. Furthermore, *Xenocyprioides* was established by Chen (1982) in Guangxi Province, China, but it is similar to Danioninae in morphological characteristics (Liu, 1998) and not closely related to other genera of *Xenocyprinae* based on phylogenetic analyses (Fang et al., 2009; Xiao et al., 2012) and differences in habitats and habits. Therefore, *Xenocyprioides* were not included in the present study.

Based on the results of the aforementioned molecular studies, there is still ambiguity regarding the phylogenetic positions and evolutionary history of some species, and the systematic questions have not been adequately answered after decades of discussion. For example, the genus status of *D. hupeinensis* remains controversial. Based on its morphology, early development, and substitution of pharyngeal teeth, Shan (1998) proposed the movement of *D. hupeinensis* from *Distoechodon* to *Xenocypris* and renamed it as *X. hupeinensis.* Phylogenetic analysis from mitochondrial evidence supports this point of view (Zhang et al., 2022). However, the current taxonomic characteristics between genera of *Xenocyprinae* were in accordance with the number of rows of lower pharyngeal teeth and folds of the pelvic fin base still supported it as the *Distoechodon* genus (Liu, 1998). Another controversy is whether *Plagiognathops* is a valid genus. Based on the three rows of pharyngeal teeth, *X. microlepis* was classified into the genus *Xenocypris* (Liu, 1998). However, Zhao et al. (2009) reclassified it as *Plagiognathops* because the lateral line scales were more than 70. Both views are supported by different morphological evidence and phylogenetic studies based on different molecular markers that show different topologies (He et al., 2004; Xiao et al., 2012; Zhang et al., 2022). Moreover, it is still debated whether *D. compressus* is a valid species in both morphological and molecular studies (support as a valid species: Yang, 1964; Cao and Meng, 1992; Liu, 2002a; Liu et al., 2021; Zhao et al., 2009; seem as just a subspecies: Bănărescu, 1970; He et al., 1989; Liu, 1998; Xiao et al., 2012). Although there is growing evidence to support the validity of *D. compressus* (Lin et al., 2020; Chen et al., 2021), there are no systematic characteristics or methods for classification, and even common molecular markers cannot distinguish it from its related species (*D. tumirostris*) (Zhang et al., 2022). Therefore, the direct aim of this study was to understand the validity of *D. compressus* and to further identify the variation in mitogenomes within *Xenocyprinae*.

Mitochondrial DNA (mtDNA) is a closed-circular molecule found in most animals and is independent of the nuclear genome. Animal mtDNA is generally small (15–20 kb) and contains 37 genes, 13 protein-coding genes (PCGs), 22 tRNAs, and 2 rRNAs (Macey et al., 1997; Satoh et al., 2016; Kobayashi et al., 2021). Mitochondria play an essential role in oxidative phosphorylation, which is essential for the production of ATP and a variety of other biochemical functions (Chaban et al., 2014). Mitochondrial DNA has the advantages of matrilineal inheritance and evolutionary conservation and has been used as a genetic marker to detect genetic differentiation and molecular evolution in population genetics (Avise, 2000; Miya and Nishida, 2015). Notably, the complete mtDNA genome has been proven to be more informative at deep phylogenetic levels (Kong et al., 2020; Wang et al., 2021) and is useful in recovering internal nodes with high statistical support compared to partial mtDNA genes (Yu et al., 2022). With rapid advances in sequencing technologies, complete mitogenome sequences are becoming available, providing abundant data on comparative analyses of mitogenomes and phylogenetic reconstruction (Shi et al., 2018). From single conservative protein-coding genes, such as *Cox1* and Cyt *b*, to multi-gene combination, phylogenetic studies have entered the omics era based on complete mitochondrial genomes or even the whole genomes (Ellegren, 2014; Koepfli et al., 2015). Phylogenetic analyses based on mitogenome sequences often result in better resolution than analyses that use few gene sequences (Bleidorn et al., 2009; Kobayashi et al., 2021). At present, except *D. compressus* and *D. hupeinensis*, the complete mitochondrial genomes of other species of the subfamily *Xenocyprinae* have been reported (Saitoh et al., 2006; He et al., 2008; Liu et al., 2014; Hu et al., 2015a, 2015b; Xu et al., 2019; Xue et al., 2019; Liu et al., 2021). The same phylogenetic results were analyzed by complete mitogenomes, and *X. fangi* clustered with the genus *Distoechodon* (Liu et al., 2021). Hence, the phylogenetic relationships and speciation within the subfamily *Xenocyprinae* need further discussion.

In the present study, we systematically reviewed the description and taxonomic history of the subfamily *Xenocyprinae* (in Abstract). We then sequenced the complete mitogenome of *D. compressus* and compared the mitogenomes of *Xenocyprinae* to reconstruct the phylogenetic relationship and estimate divergence time among the subfamily *Xenocyprinae*. The aim of this study was to understand the phylogenetic relationships and evolutionary history of the subfamily *Xenocyprinae*, confirm the genus classification and the validity of *D. compressus*, and provide references for the systematics of cyprinid fish.

## Materials and methods

### Sampling, DNA extraction, polymerase chain reaction, and sequencing

Specimens were collected using gill nets from a tributary of the Ting River (116.62°E, 25.35°N) in Liancheng County, Fujian Province, China, from April to July 2021. Thirty specimens were identified following Zhao et al. (2009). Muscle tissues were preserved in 95% ethanol and stored at the Institute of Oceanology, Minjiang University. Total genomic DNA was extracted using the salt extraction method (Aljanabi and Martinez, 1997). The complete mitochondrial genome of *D. compressus* was amplified based on the two-step process (six universal primers and six specific primers described in Supplementary Table S1). Polymerase chain reaction (PCR) amplification was carried out in a 20 μL reaction following Zhang et al. (2021). The PCR products were examined using electrophoresis on a 1.0% TAE agarose gel and sequenced by Sangon Biotech (Shanghai, China).

### Annotation, gene order, and genomic structure analyses

The overlapping fragments were assembled into a linear mitochondrial DNA sequence using SeqMan (DNASTAR), and the assembled sequences were manually checked (Jin and Sun, 2018). The complete mitochondrial genome sequences of *Xenocyprinae* species included all the Xenocyprinae fish that have been reported so far, representing four genera (species and accession numbers are listed in Table 1), except for *Xenocyprioides*. The complete mitochondrial genome sequences were downloaded from the GenBank with their features and structures. All the sequences were annotated using the MITOS web server (Bernt et al., 2013) and determined in MitoFish (http://mitofish.aori.utokyo.ac.jp/) to compare the genomic structure and gene order (Sato et al., 2018). Transferred RNA genes and their secondary structures were predicted using the tRNAscan SE web server (http://lowelab.ucsc.edu/tRNAscan-SE/) (Lowe and Chan, 2016). The composition of amino acids, nucleotide bases, and relative synonymous codon usage (RSCU) was calculated using MEGA X software (Kumar et al., 2018). Variable sites among the sequences were also detected using MEGA software. Nucleotide composition skew was calculated using the following formulas: AT-skew = (A—T)/(A + T) and GC-skew = (G—C)/(G + C) (Perna and Kocher 1995).

**TABLE 1:** Comparison of size and base composition of mitogenomes.

Species	Length	A + T content	AT-skew	GC-skew	PCGs AT-skew	PCGs GC-skew	CR size	Reference/GenBank accession number
*Distoechodon tumirostris*	16620	56.8	0.106	−0.26	0.039	−0.29	937	DQ026431, He et al. (2008); NC011208
*Distoechodon compressus*	16621	56.8	0.106	−0.26	0.039	−0.29	937	In this study
*Xenocypris fangi*	16619	56.8	0.104	−0.26	0.035	−0.28	935	MW366639, Liu et al. (2021)
*Xenocypris* argentea	16629	56.5	0.104	−0.26	0.032	−0.29	934	AP009059, Saitoh et al. (2006)
*Xenocypris davidi*	16630	56.7	0.104	−0.25	0.032	−0.29	935	KF039718, Liu et al. (2014); MN264265, Xu et al. (2019)
*Xenocypris yunnanensis*	16630	56.7	0.104	−0.26	0.032	−0.29	934	KY993905, Xu et al. (2019)
*Plagiognathops microlepis*	16623	56.8	0.097	−0.24	0.025	−0.27	938	KF383387, Hu et al. (2015a)
*Pseudobrama simoni*	16618	56.8	0.075	−0.24	0.005	−0.26	939	KF537571, Hu et al. (2015b)

### Phylogenetic analysis

Phylogenetic analyses were reconstructed using maximum likelihood (ML) and Bayesian inference (BI) methods, based on concatenated nucleotide sequences of 13 PCGs from 12 individuals belonging to the subfamily *Xenocyprinae*, with *C. carpio*, *H. molitrix*, and *A. nobilis* as outgroups. The best-fit partition model of nucleotide evolution of PCGs was identified using PartitionFinder v2 (Lanfear et al., 2012) in the PhyloSuite platform (Zhang et al., 2020), and it was GTR + I + G according to the Akaike information criterion (Bozdogan 1987). ML and BI analyses were performed using MrBayes v3.2.7 and RaxML v8.2.12 programs following their manuals, respectively (Ronquist et al., 2012; Stamatakis 2014). Bootstrap ML analysis was implemented under the GTRGAMMAI model, and 1000 replications were used to evaluate the bootstrap support values and search for the best ML tree. BI analysis was run as two simultaneous Markov chain Monte Carlo (MCMC) chains for 10 million generations, with sampling of every 1000 generations, using a burn-in rate of 25%. Phylogenetic trees were visualized through the online tool Interactive Tree Of Life v5 (iTOL, https://itol.embl.de/) (Letunic and Bork 2021).

### Divergence time estimation

Divergence time among the subfamilies was estimated using the amino acid sequences of 13 PCGs with a relaxed clock log-normal model in BEAST v.1.10 (Drummond and Rambaut, 2007). A model of the Yule process was selected for the tree prior. To estimate the divergence time calibration, two calibration points were used as priors for the divergence time of the corresponding splits. The most recent common ancestor of *Xenocyprinae* is dated to 15 million years ago (Mya), as described by Chang et al. (1996) and Chen et al. (2005). Also, the 18.80 Mya point calibration was set as the divergence time of *Hypophthalmichthyinae* from cyprinids (Wang et al., 2012). The final Markov chain was run twice for 100 million generations, with sampling of every 10,000 generations. The first 10% of generations were discarded as burn-in, according to the convergence of chains checked using Tracer v. 1.7, and the ESS values of all parameters were above 200. The maximum clade credibility tree was generated using TreeAnnotator v. 2.4.1 (part of the Beast package) and visualized in the iTOL web tool.

## Results and Discussion

### Mitogenome structure and organization

**FIGURE 1:**
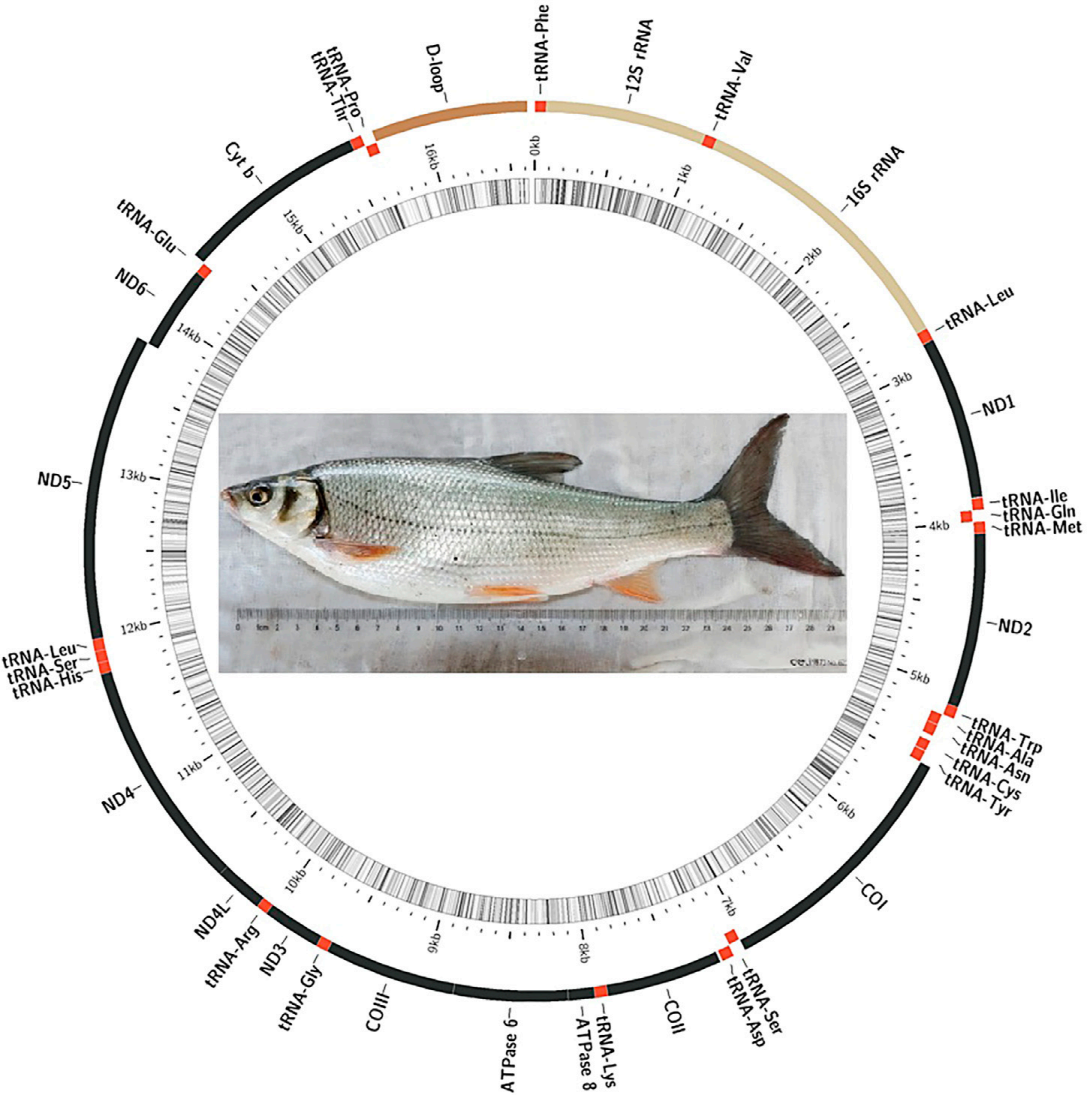
Mitogenomic map of the mitogenomes of *Xenocyprinae* species.

In the present study, the complete mitogenome of *D. compressus* was successfully sequenced and annotated. It was deposited in the GenBank under accession number OM994436. All the reported mitogenomes of *Xenocyprinae* were compared to analyze the similarities and differences in structure and organization between the sequences. The mitogenomes of nine xenocyprinid fish, ranging from 16623 bp to 16630 bp length, have 2511 variable sites, where 698 variable sites are singletons among them. The gene content and order were as expected for a typical vertebrate mitogenome which comprises 13 PCGs, 22 tRNA genes, 2 rRNA genes, and 1 non-coding control region. More than half of the genes were encoded on the heavy (H-) strand: 9 PCGs and 14 tRNAs (Figure 1). The bias toward high AT content (55.8%–58.8%) is consistent with general findings in teleost mitogenomes (Sharma et al., 2020; Sun et al., 2021; Yu et al., 2021). The average nucleotide composition of all sequences is A (31.2%), T (25.5%), G (16.2%), and C (27.1%). For the complete mitogenomes, AT-skew was all positive, ranging from 0.075 (*P. simoni*) to 0.106 (*D. compressus* and *D. tumirostris*), and GC-skew was slightly negative (−0.24 ∼ −0.26) (Table 1).

### Protein-coding genes

Similar to other vertebrates, ATG is the regular initiation codon in almost all PCGs, except for *Cox1*, which uses GTG. However, many PCGs were not terminated with TAG or TAA but possessed an incomplete stop codon (six or seven genes), which was more than most of the mitogenomes of teleosts (Sun et al., 2021; Yu et al., 2021; Zhang et al., 2021). This can be corrected by posttranscriptional polyadenylation (Jondeung et al., 2007; Shi et al., 2018). Ranging from 11422 to 11429 bp, there was a slightly positive AT-skew (0.005–0.039) and negative GC-skew (−0.26 ∼ −0.29) (Table 1). This is consistent with the bias of using A rather than T in the PCGs of most teleosts, suggesting that there was no unique selective pressure or processes of the xenocyprinid species detected in several fish (Yu et al., 2021; Zhang et al., 2021). Amino acid codon usage of 11 mitogenomes was assessed by relative synonymous codon usage (RSCU) values. Leucine 2 (Leu2), threonine (Thr), and proline (Pro) were the most frequently translated amino acids, whereas glutamine (Glu) and aspartate (Asp) were the least used amino acids. CUA (Leu), AUU (Ile), and AAU (Asn) were codons commonly used in *Xenocyprinae* mitogenomes. The over-usage of A and T at the third position indicates the possibility of genome bias, optimal selection of tRNA, or DNA repair efficiency, referring to other teleosts (Fischer et al., 2013; Zhang et al., 2021).

### Non-coding genes

The sizes of the 22 tRNA genes ranged from 68 bp to 76 bp, which comprised 9.4% of the complete mitogenomes. Most secondary structures of tRNAs were recovered as ordinal clover-leaf structures (21 of the 22 tRNAs), which included the amino acid-accepting stem (AAS), dihydrouridine stem and loop (DSL), anticodon stem and loop (ASL), thymidine stem and loop (TSL), and variable stem and loop (VSL). However, tRNASer (AGN) lacks the DHU arm, which is commonly observed in many metazoan mitogenomes (Frazen-Abel and Hagerman, 2008; Watanabe et al., 2014). The two rRNA genes were separated by tRNAVal, with lengths of 961–964 bp and 1688–1693 bp. Similarly, a strong AT-bias was also detected in rRNAs, with a strongly positive AT-skew (0.253–0.269) and a slightly negative GC-skew (−0.06 ∼ −0.08).

The length of the CR ranged from 930 to 938 bp, with a significantly higher A + T content (65.5%) and relatively low AT-skew (0.0046). The control region contains three domains: a termination-associated sequence (TAS), central conserved domain (CD), and conserved sequence block (CSB). The identified conserved sequences also exist in other Cypriniformes (Liu, 2002b; Xiao and Zhang, 2000; Shi et al., 2018). Miya and Nishida (2015) proposed that CR plays a crucial role in the initiation and regulation of transcription and replication. The TAS may terminate the synthesis of the heavy strand (Fischer et al., 2013), and the central conserved domains (CSB-F, CSB-E, and CSB-D) are thought to be associated with the positioning of RNA polymerase for both priming replication and transcription (Castresana, 2000). All the conserved blocks may maintain the function of the control region. We found that CSB2 was the most conserved. However, some domains (such as CSB-E) experienced rapid evolution 5 to 10 times that of the complete mtDNA. In this study, the variation in CR may be an important marker for distinguishing different species of *Xenocyprinae*.

### Genetic distance and variation in mitochondrial sequences

The pairwise genetic distances ranged from 0.002 (*X. davidi* and *X. argentea*) to 0.104 (*P. microlepis* and *P. simoni*) with an average of 0.064 ± 0.029 (Table 2). In the present study, the variation sites between the two *Distoechodon* species were only 0.28% of the complete mitogenomes, far less than other intra-species studies. However, morphological differences between these two *Distoechodon* species have been widely reported (Cao and Meng, 1992; Zhao et al., 2009; Lin, 2020; Chen et al., 2021). The amount of nucleotide variation in the mitogenomes that can be used to distinguish species or genera levels is still not clarified. The main differences in nucleotide composition included two parts. First, the length of each part showed three differences in 16s rRNA, *ND2*, and the gaps after *ND2*. The length of the 16 S rRNA ranged from 1688 bp to 1693 bp. *ND2*, *X. davidi*, *X. argentea*, and *X. yunnanensis* had a complete stop codon (TAA), whereas the remaining sequences had an incomplete stop codon (T++). Six nucleotides were identified between *ND2* and tRNA^Trp^ in *X. davidi*, *X. argentea*, and *X. yunnanensis* more than others. Hence, these two differences can be used as loci to distinguish *Xenocypris* from other genera. In addition, we examined the number and percentage of variation in all PCGs. *ND2* and *ND4* were the most conserved PCGs, with variation sites accounting for 5%. Alternatively, the variation sites of *ND1*, *ATP6*, *ND3*, *ND5*, and *ND6* exceeded 20% among the nine xenocyprinid fish. *CR* also showed a high variation within less than 1 kb sequence, but most of the variation sites were concentrated in the *CD* and *CSB* domains.

**TABLE 2:** Pairwise genetic distance between each two species within *Xenocyprinae*.

Species	Dc	Dt	Xf	Xa	Xd	Xy	Pm
*Distoechodon compressus*							
*Distoechodon tumirostris*	0.0028						
*Xenocypris fangi*	0.0209	0.0205					
*Xenocypris argentea*	0.0672	0.0664	0.067				
*Xenocypris davidi*	0.0672	0.0664	0.0669	0.0024			
*Xenocypris yunnanensis*	0.0668	0.0658	0.0663	0.0126	0.011		
*Plagiognathops microlepis*	0.0844	0.0838	0.0844	0.0809	0.0807	0.0811	
*Pseudobrama simoni*	0.0832	0.0829	0.0842	0.0873	0.0871	0.0864	0.1039

### Phylogenetic relationship and updated classification of the subfamily *Xenocyprinae*

**FIGURE 2:**
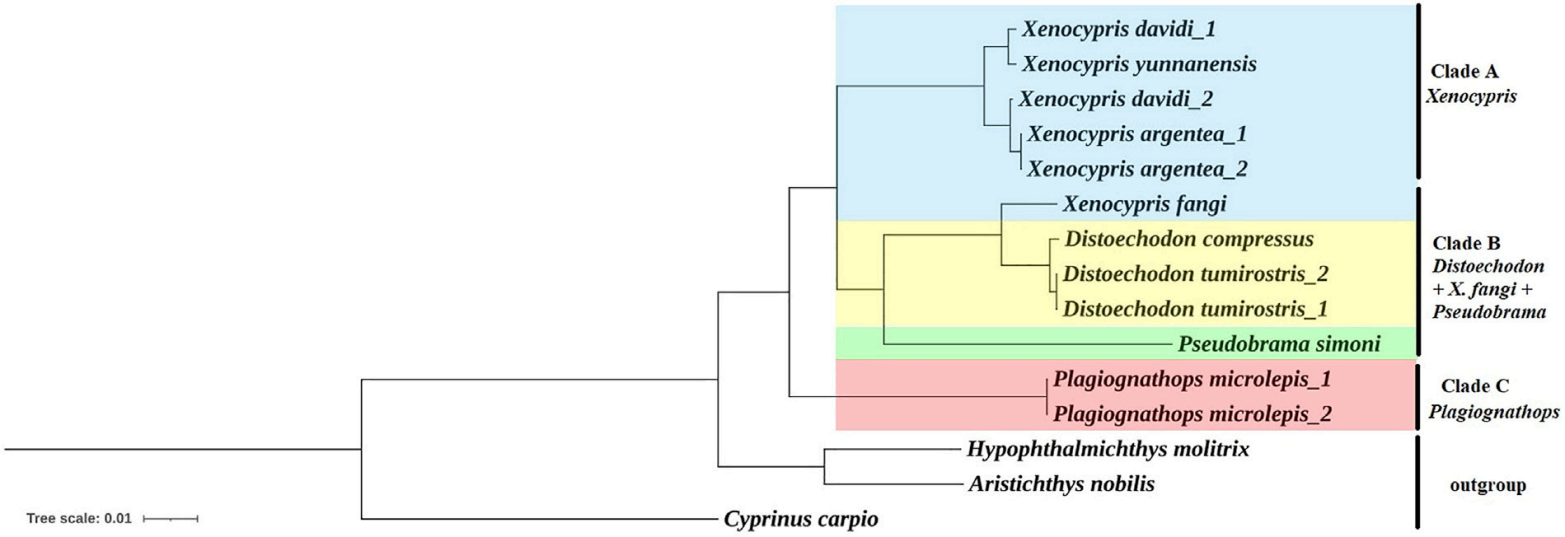
Phylogenetic relationship within the subfamily *Xenocyprinae* using BI and ML methods based on the 13 PCGs. Note: All the support values for the ML/Bayesian method are 1.00 or 100 so that it is not labeled at the nodes in the figure.

Phylogenetic trees were constructed using the BI and ML methods, based on concatenated nucleotide sequences of 13 PCGs. The phylogenetic trees exhibited a congruent topology according to both methods. As shown in Figure 2, the subfamily *Xenocyprinae* was monophyletic within Cyprinidae corroborating the result of previous studies on the phylogeny of Cyprinidae (Saitoh et al., 2006; Wang et al., 2007; He et al., 2008; Yang et al., 2015; Tao et al., 2019; Cheng et al., 2022). Within the subfamily *Xenocyprinae*, there were three clades representing different genera and their phylogenetic relationships. *Plagiognathops* was located at the base of the phylogenetic tree with a pairwise genetic distance of more than 0.08 between all the other xenocyprinid fish, which are completely different from those of previous studies based on partial mitochondrial genes (He et al., 2004; Xiao et al., 2012; Cheng et al., 2022). For the remaining two clades, it mainly consisted of genera *Xenocypris* and (*Distoechodon + Pseudobrama*). *X. fangi* is closely related to *Distoechodon* with a genetic distance of less than 0.02. We suspect that sampling or sequencing in Liu et al. (2021) contributed to this unusual result. All previous phylogenetic analyses using partial mtDNA genes revealed that *X. fangi* clustered to the *Xenocypris* clade (He et al., 2004; Xiao et al., 2012; Zhang et al., 2022)*.* Therefore, more sequences of *X. fangi* are needed to understand their accurate phylogenetic position.

The current results clarified some controversies in the systematics of the subfamily *Xenocyprinae*. All of the aforementioned results support the validity of genus *Plagiognathops*. The key characteristics of *Plagiognathops* are lateral line scales of less than 50 and gill rakers on the first gill arch of more than 100 (Zhao et al., 2009). Second, strong evidence supports the validity of *D. compressus*. Previous studies based on partial mitochondrial markers could not accurately distinguish *D. compressus* and *D. tumirostris* (Cheng et al., 2022; Zhang et al., 2022). In the present study, using complete mitogenomes, the two species could be separated from each other by phylogenetic analysis. Although the genetic distance between the two species is very low, we also found the main variation between two *Distoechodon* species in *Cox1* and *ND5* genes, so that species classification can be identified by specific loci.

### Divergence time estimation

**FIGURE 3:**
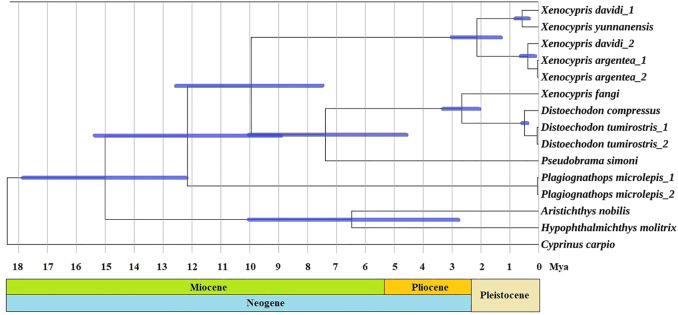
Divergence time estimation of *Xenocyprinae* utilized two fossil clocks in BEAST based on the best-scoring maximum-likelihood tree. Note: Node bars show 95% *CI*s of the divergence time estimate.

The two clusters (*Xenocypris* and (*Distoechodon + Pseudobrama*)) diverged 10.0 Mya (95% CI: 7.4–12.6 Mya) (Figure 3). The divergence time in this study was earlier than that of previous studies on cyprinid fish, which were based on nuclear recombination activating gene 2 and the mitochondrial 16 S rRNA and cytochrome *b* genes (6.7 Mya, Wang et al., 2012) but later than that reported for East Asian opsariichthyin-xenocyprinin-cultrin fish (14.4 Mya) by Cheng et al. (2022). *Plagiognathops* was reconfirmed as a valid genus and began to diverge from *Xenocypris* and *Distoechodon* approximately 12.1 Mya. The two *Distoechodon* species (*D. compressus* and *D. tumirostris*) separated from each other 0.6 Mya and were still at an early stage of differentiation. Xenoprinins, as an important part of East Asian freshwater fish, were recently shown to be related to monsoon-driven climatic conditions and formation of river and river–lake environments in East Asia (Cheng et al., 2022). With a close relationship to the Tibetan Plateau uplift from 25 to 20 Mya (Xiao et al., 2012), the Asian monsoon climate was rebuilt in the Early Neogene (23 Mya) (Guo et al., 2002; Favre et al., 2015). The East Asian monsoon was further strengthened in the late Early Miocene, from the Middle Miocene (approximately 15–13 Mya) to the Late Miocene (approximately 10–7 Mya), and the Middle Pliocene (approximately 3.5 Mya) (Clift et al., 2008). In this study, the major radiation of *Xenocyprinae* species possibly occurred in the Middle to Late Miocene and Late Pliocene, which also confirmed that Xenocyprinae fish were the vital case of Asian monsoon climate resulting in speciation and diversification.

## Data Availability

The mitochondrial genomes for this study can be found in the GenBank with accession number OM994436.
