# Supplementary material for: Comparative mitogenomes provide new insights into phylogeny and taxonomy of the subfamily Xenocyprinae (Cypriniformes: Cyprinidae)
Source: Front Genet. 2022 Oct 25;13:966633. doi: 10.3389/fgene.2022.966633 (PMC9640767; doi:10.3389/fgene.2022.966633)
Supplement: Supplementary file 1 [file Table1.DOCX]

Supplementary Material

## Table S1. Information of primers in this study

| Primer name | Forward sequence (5′→3′) | Reverse sequence (5′→3′) | Annealing temperature (°C) | Product size(bp) |
| --- | --- | --- | --- | --- |
| Partial PCRs (universal primers) | | | |  |
| 12S | TACACATGCAAGTCTCCGCA | ACTTACCGTGTTACGACTTGCCTC | 57 |  |
| 16s | CGCCTGTTTATCAAAAACAT | CCGGTCTGAACTCAGATCACGT | 53 |  |
| ND2 | CACCACCCWCGAGCAGTTGA | CGKAGRTAGAAGTAHAGGCT | 52 |  |
| CO1 | TCAACCAACCACAAAGACATTGGCAC | TAGACTTCTGGGTGGCCAAAGAATCA | 55 |  |
| CO3 | TTCTGAGCCTTCTAYCA | CAAGACKGKGTGATTGGAAG | 52 |  |
| CYTB | GCCAAWGCTGCWGAATAMGCAAA | GGTGGCKCCTCAGAAGGACATTTGKCCTCA | 52 |  |
| Long PCRs (specific primers) | | | |  |
| Gap1 | GTCACTCTCCCCTGTCAAAA | CGTTGAACCGTAGTCACTG | 54 | 1172 |
| Gap2 | CCGCTATTAAGGGTTCGTTT | TGTCTCATTCCCCTGTGATT | 53 | 1744 |
| Gap3 | ACAATTATAGCCTTAGCCGC | AATTCGGCTCGAATGAGAAG | 53 | 719 |
| Gap4 | AGGAGGGGACCCAATTCTAT | ACCCCTGATGCTAGTAGAAC | 54 | 3076 |
| Gap5 | CCACTTTACATCCGAACACC | AGTTGGTTATGGGGGTTCTT | 54 | 4712 |
| Gap6 | CATTCCGTCCAATCACTCAA | GGCTAAAATTTGTGCCTGATG | 53 | 1472 |
